# New Jersey Medical Institute, Located at Burlington, N. J.

**Published:** 1853-01

**Authors:** 


*New Jersey Medical Institute, located at Burlington, N. J.*—The advantages offered to students of medicine during the summer months by the above institution, are sixteen lectures a week, from the first Monday in April to the first of August, on the various branches taught in the schools, together with hospital instruction in Philadelphia, without the necessity of a residence there, and without additional expense—free tickets to and from one place to the other being furnished twice a week. From the the first of September to the commencement of the winter college terms, the instruction will consist of clinics, and examinations embracing a critical review of the previous course. A course on practical pharmacy is also given.

The teachers in the institute, Drs. J. B. Coleman, Joseph Parish, Zachariah Read, Franklin Gauntt, Silas S. Brooks, S. W. Butler and H. Rand, are known to the medical profession as men of ability. The expense of the course is sixty dollars.

